# Tight junction protein claudin-1 is a novel internalization factor for swine enteric coronaviruses infection

**DOI:** 10.1128/mbio.03496-25

**Published:** 2026-01-09

**Authors:** Zhongyuan Li, Jianfei Chen, Yunyan Chen, Shouping Hu, Huan Li, Liang Li, Mei Xue, Li Feng

**Affiliations:** 1 State Key Laboratory of Animal Disease Control and Prevention, Harbin Veterinary Research Institute, Chinese Academy of Agricultural Sciences 687216 , Harbin, Heilongjiang, China; The University of Iowa, Iowa City, Iowa, USA

**Keywords:** coronavirus, claudin-1, internalization, spike protein

## Abstract

Swine enteric coronaviruses (SeCoVs), including transmissible gastroenteritis virus (TGEV), porcine epidemic diarrhea virus (PEDV), and porcine deltacoronavirus (PDCoV), have been reported to use aminopeptidase N (APN) as a cellular receptor. However, APN alone cannot effectively explain the infection of both APN-positive and APN-negative enterocytes by PEDV and TGEV, nor the wide host range of PDCoV, suggesting the involvement of other host factors. In this study, we demonstrate that TGEV infection in piglets upregulates claudin-1 expression not only in infected cells but also in uninfected cells. Claudin-1 levels correlated strongly with TGEV N protein levels in the jejunum of infected piglets. Functional studies revealed that claudin-1 overexpression enhanced cellular susceptibility to TGEV, PEDV, and PDCoV, whereas its knockout significantly attenuated infection. Mechanistically, claudin-1 specifically interacts with the S1 or receptor-binding domain (RBD) of SeCoVs and promotes viral internalization. Furthermore, induction of claudin-1 in piglets promotes PDCoV infection in the intestine. Notably, claudin-1 also binds to the S1 protein of severe acute respiratory syndrome coronavirus 2 (SARS-CoV-2). Collectively, our results identify claudin-1 as a novel internalization factor for porcine enteric coronaviruses, playing a critical role in facilitating infection within the digestive tract, and highlight its potential as a target for future clinical interventions.

**IMPORTANCE:**

We observed a downregulation in the expression of the majority of tight junction proteins in intestinal tissues infected with transmissible gastroenteritis virus (TGEV). However, unexpectedly, claudin-1 exhibited a significant upregulation in intestinal epithelial cells. This intriguing finding prompted us to delve deeper into the potential role of claudin-1 in facilitating virus invasion of epithelial cells. Utilizing overexpression and knockout cell lines, we demonstrate that claudin-1 is an internalization factor for swine enteric coronaviruses (SeCoVs), including TGEV, porcine epidemic diarrhea virus (PEDV), and porcine deltacoronavirus (PDCoV). Notably, claudin-1 interacts with the S1 protein of TGEV, PEDV, PDCoV, and severe acute respiratory syndrome coronavirus 2 (SARS-CoV-2), spanning across alpha, beta, and delta coronaviruses. Our findings provide deeper insights into the infection mechanisms and pathogenesis of SeCoVs and SARS-CoV-2.

## INTRODUCTION

Coronavirus poses a serious threat to human and animal health (1). Severe acute respiratory syndrome coronavirus 2 (SARS-CoV-2), belonging to the Betacoronavirus, was identified as the causative pathogen of coronavirus disease 2019 (2). The alphacoronavirus-transmissible gastroenteritis virus (TGEV) and porcine epidemic diarrhea virus (PEDV) and the deltacoronavirus-porcine deltacoronavirus (PDCoV) are highly infectious intestinal pathogenic viruses that are the main pathogens of fatal watery diarrhea in piglets, causing significant economic losses to the pork industry (3, 4). Currently, PDCoV is a concern due to its broad host range, including humans (5). Chickens, turkey poults, and mice can be experimentally infected by PDCoV (6–8).

Receptor interaction plays a key role in the cell and tissue tropism of coronaviruses, as well as in pathogenesis and cross-species transmission (9). Many viruses are known to use multiple receptors in parallel or in series (10, 11). The requirement for aminopeptidase N (APN) as a receptor for TGEV, but not for PEDV and PDCoV, is well established (12–15). The African green monkey Vero cell lines historically used for isolating and propagating PEDV strains do not express APN, indicating that other receptors may be involved in PEDV entering these cells (16, 17). PDCoV utilizes pAPN as a primary receptor for virus attachment, but the presence of a second co-receptor contributes to the permissiveness of cells for infection. Furthermore, the PDCoV co-receptor can retain function independent of pAPN (18). Therefore, unraveling the APN-dependent or independent receptors of swine enteric coronaviruses (SeCoVs) is crucial not only for enhancing our understanding but also for pinpointing novel therapeutic targets for recurrent coronavirus diseases.

The infection caused by SeCoVs compromises the integrity of the intestinal barrier and disrupts intestinal homeostasis (19). Integrity of the intestinal epithelium is maintained mainly by tight junctions (TJs) and adherens junctions (20). In many kinds of endothelial and epithelial cells, the TJ complex is the most tightly connected complex (21, 22). The resistance of piglets to SeCoV infection and the integrity of the intestinal mucosal barrier are significantly affected by some tight junction proteins, especially occludin and claudins (23, 24). Claudins have a similar membrane topology of four transmembrane domains, with cytosolic amino and carboxy termini, forming two extracellular loops (ECLs), ECL1 and ECL2 (25, 26). According to recent research, claudins are crucial host factors for the entrance of several viruses, including the human hepatitis C virus (HCV) and the dengue virus (27–30). Or by degrading TJs to facilitate viral entry and dissemination, such as West Nile virus, Japanese encephalitis virus, norovirus, human rhinovirus, and porcine reproductive and respiratory syndrome virus (31–35). Whether claudins are the internalization factors that mediate entry of TGEV and other enteroviruses remains to be determined.

In this study, we found that TGEV infection significantly prompts the expression of claudin-1. In an attempt to define the role of claudin-1 in SeCoVs’ entry, we performed co-immunoprecipitation studies and demonstrated that overexpressed claudin-1 could interact with the S1 proteins of TGEV, PEDV, PDCoV, and SARS-CoV-2 and is important for the internalization of SeCoVs. Finally, lubiprostone treatment induced the expression of claudin-1 in piglets; as a result, the treated cells showed increased susceptibility to PDCoV infection. These results suggest that claudin-1 is an internalization factor for SeCoVs, providing a new direction for the development of antiviral drugs against SeCoV infections.

## RESULTS

### TGEV infection significantly increased claudin-1 expression in cells

**Fig 1:**
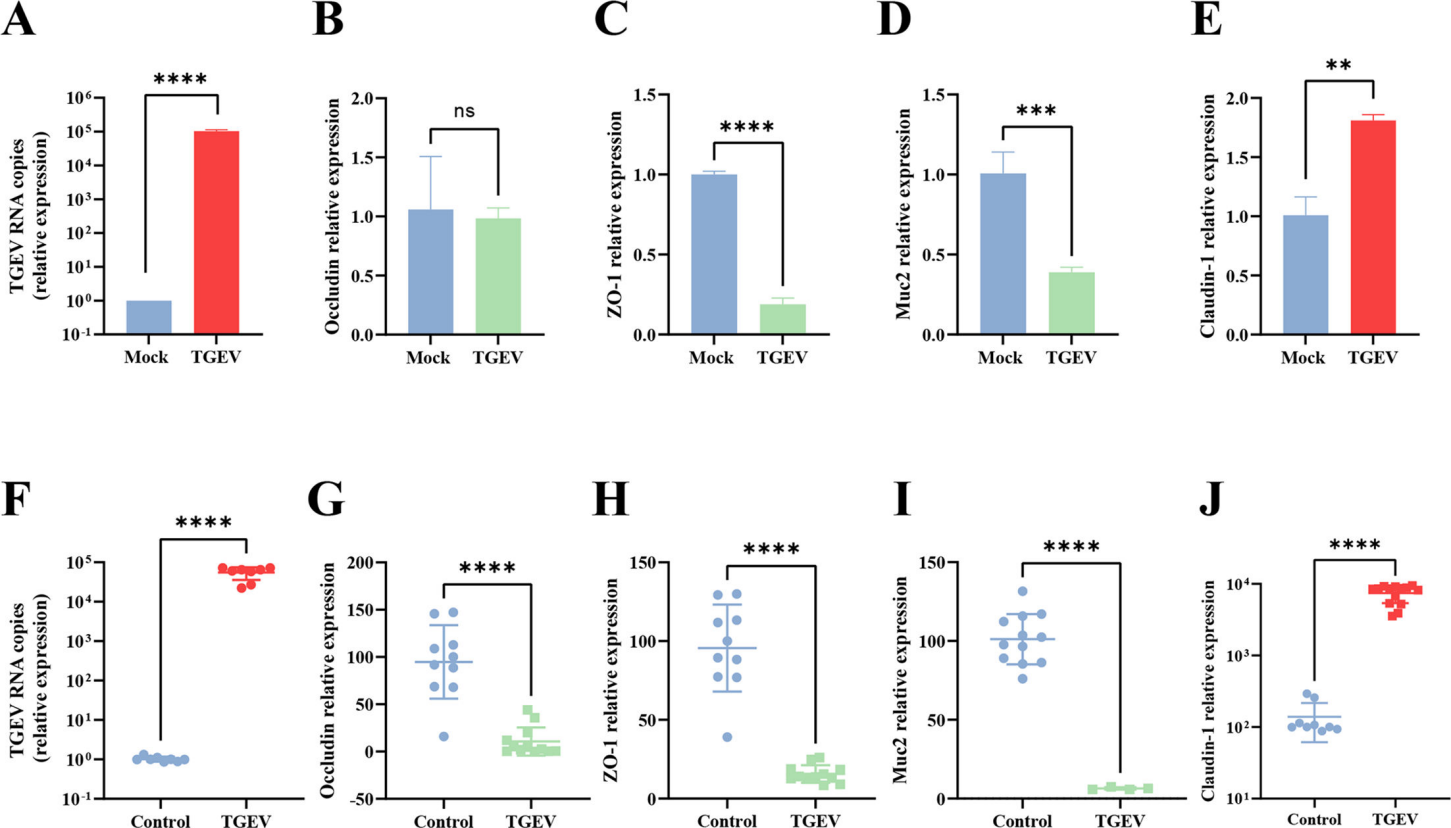
TGEV infection increases the expression of *claudin-1* in IPI-FX cells and intestinal tissues of piglets. (**A**) The TGEV RNA copies were determined by reverse transcription-quantitative polymerase chain reaction (RT-qPCR). (**B–E**) IPI-FX cells were infected with TGEV H165 (multiplicity of infection [MOI] = 0.1) for 24 hpi. The relative mRNA expression of *occludin* (**B**), *ZO-1* (**C**), *Muc2* (**D**), and *claudin-1* (**E**) was detected by RT-qPCR with the indicated gene primers. (**F**) The TGEV RNA copies in the intestinal tissues of piglets inoculated with TGEV were determined by RT-qPCR. (**G–J**) The relative mRNA levels of *occludin* (**G**), *ZO-1* (**H**), *Muc2* (**I**), and *claudin-1* (**J**) were assessed by RT-qPCR. Statistical analyses were performed using GraphPad Prism 9 and Student’s *t*-test. All tests were carried out in triplicate. The error bars show the standard deviations from three experiments. Significant differences: ****P < 0.0001, ***P < 0.001, **P < 0.01; ns, not significant.

Tight junction proteins are crucial for preserving the intestinal mucosal barrier’s integrity and serve as entry points for certain viruses. To elucidate the interplay between tight junction protein gene expression and TGEV infection, we compared the expression of *claudin-1*, *ZO-1*, *occludin*, and *Muc2* genes in TGEV-infected porcine intestinal epithelial cells (IPI-FX) with that of a control group. As shown in Fig. 1A, the viral RNA copy number reached 10^5^ in TGEV-infected IPI-FX cells, indicating the efficient replication of TGEV in these cells. Our analysis revealed no significant alterations in *occludin* expression, while the overall expression of *ZO-1* and *Muc2* displayed a downward trend; particularly notable was the significant increase (*P* < 0.01) in *claudin-1* gene expression (Fig. 1B through E). These data suggest that TGEV infection enhances claudin-1 expression *in vitro*.

To further explore the expression of tight junction proteins in pigs infected with TGEV, we employed RT-qPCR to analyze the expression of tight junction genes in 3-day-old piglets infected with TGEV and normal piglets. The viral RNA copy number reached 10^4^ to 10^5^ in TGEV-infected jejunum, indicating the efficient replication of TGEV *in vivo* (Fig. 1F). Notably, the expression of *ZO-1*, *occludin,* and *Muc2* in the intestinal tissue of TGEV-infected piglets was significantly reduced (*P* < 0.0001) compared to healthy piglets (Fig. 1G through I). Conversely, the expression of *claudin-1* in the ileum was significantly elevated (*P* < 0.0001) in infected piglets (Fig. 1J).

**Fig 2:**
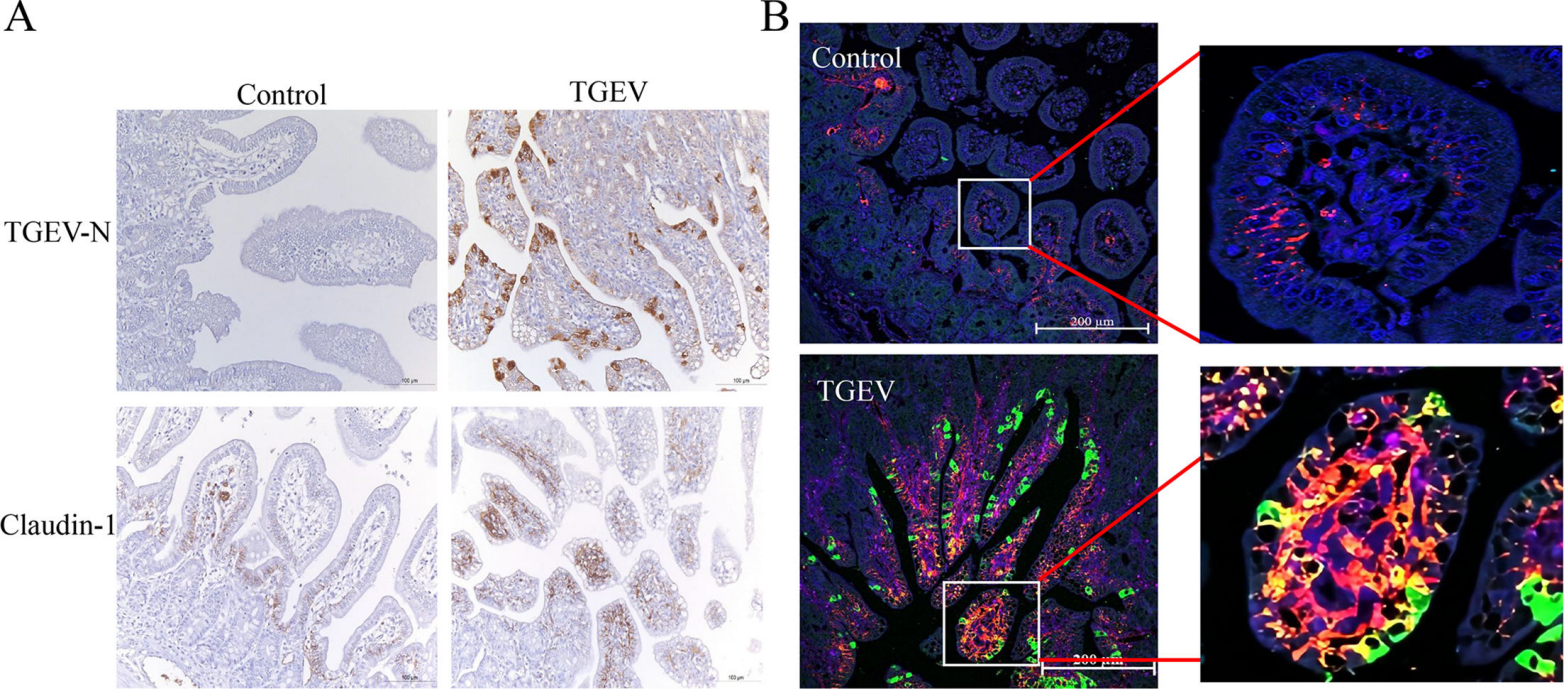
TGEV infection significantly promotes claudin-1 expression *in vivo*. (**A**) Immunohistochemical analysis was conducted to detect the expression of TGEV N protein and claudin-1 in jejunum tissues (bars, 100 μm). (**B**) Double immunofluorescence staining was performed on the jejunum tissues of TGEV-infected piglets to visualize the endogenous expression of claudin-1 and the presence of TGEV infection. Sections were incubated with primary antibodies against claudin-1 (red) and TGEV N protein (green), followed by probing with the appropriate secondary antibodies. Nuclei were stained with 4,6-diamidino-2-phenylindole (DAPI) (blue). Bars, 200 μm.

Furthermore, we validated our findings through immunohistochemistry on the jejunum of TGEV-infected or control piglets, confirming a significant increase in claudin-1 expression in TGEV-infected jejunum (Fig. 2A). We performed additional double immunofluorescence staining on the jejunum sections of piglets and found that TGEV infection in piglets upregulates claudin-1 expression not only in infected cells but also in uninfected cells. The claudin-1 expression level is well correlated with TGEV N level (Fig. 2B). These results indicate that TGEV infection significantly prompts claudin-1 expression *in vivo*.

### Overexpression of claudin-1 facilitates TGEV infection

**Fig 3:**
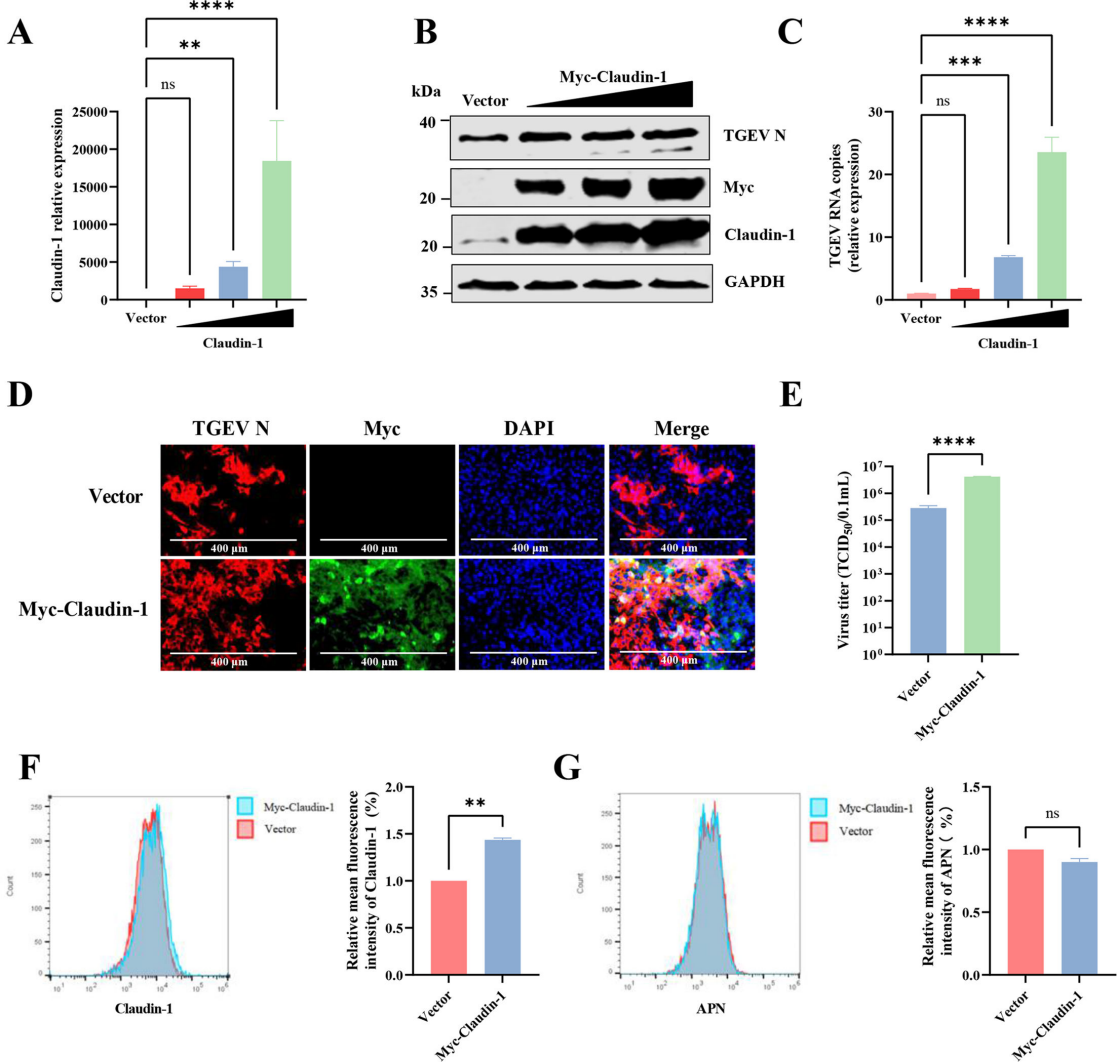
Claudin-1 facilitates the proliferation of TGEV. (**A**) IPI-FX cells were transfected with Myc-Claudin-1 plasmid (1.5, 2, and 2.5 μg) or a vector control for 24 h and infected with TGEV (MOI = 0.1) for 24 h. The mRNA expression levels of claudin-1 were detected by RT-qPCR. (**B**) The overexpression of claudin-1 and the replication of TGEV were assayed by western blotting. (**C**) TGEV RNA copies in claudin-1 overexpressed cells were detected by RT-qPCR. (**D**) The replication of TGEV in claudin-1 overexpressed cells (cells were transfected with Myc-Claudin-1 plasmid [2.5 μg]) was measured by IFA. (**E**) The virus yield was measured by a 50% tissue culture infected dose (TCID_50_) assay on ST cells. (**F and G**) Under unpermeabilized conditions, the mean fluorescence intensity (MFI) of claudin-1 (**F**) and APN (**G**) on the cell surface was detected by FC500 flow cytometer after overexpression of claudin-1. The error bars show the standard deviations from three experiments. ****P < 0.0001, ***P < 0.001, **P < 0.01; ns, not significant.

The inconsistency in the expression trends of claudin-1 with the other tight junction protein genes prompted us to determine if claudin-1 plays a role in TGEV infection. To test whether claudin-1 is needed for TGEV infection, we overexpressed claudin-1 in IPI-FX cells, which are of intestinal origin and more closely represent natural target cells during TGEV infection. We then used RT-qPCR and western blotting to quantify claudin-1 expression after transfection. Overexpression caused a 1,500–18,500-fold increase in claudin-1 expression and was confirmed by immunoblot (Fig. 3A and B). Next, we determined cell susceptibility to TGEV after claudin-1 overexpression. As expected, claudin-1 overexpression increased susceptibility to TGEV by 2- to 24-fold (Fig. 3C), suggesting a positive role of claudin-1 in TGEV infection. Consistent with the mRNA levels, western blotting using the TGEV-N monoclonal antibody showed that the replication of TGEV was significantly increased in a dose-dependent manner with the overexpression of Myc-Claudin-1 at 24 hpi (Fig. 3B). Immunofluorescence assay (IFA) revealed that Myc-claudin-1 overexpression, which substantially elevated the basal expression level of endogenous claudin-1 (Fig. S1), significantly increased the number of TGEV-infected cells by approximately threefold (Fig. 3D). Successful expression of the exogenous Myc-tagged protein was also confirmed in these cells (Fig. 3D). Cell culture supernatants were collected to determine the viral titers, and the results showed that overexpression of Myc-Claudin-1 substantially increased viral titers (Fig. 3E). In addition, we used lentiviruses encoding claudin-1 to generate a claudin-1 overexpressing IPI-FX cell line and lentiviruses that carry an empty vector to produce a control cell line. The claudin-1 overexpressing cells displayed twice as much claudin-1 on the surface as the control cell line. TGEV infection in the claudin-1 overexpressing cell line was higher than in the control cell line (Fig. S2A). To determine the subcellular localization of overexpressed claudin-1, we performed flow cytometry under non-permeabilized conditions. The results showed that cells transiently overexpressing claudin-1 and IPI-FX cells stably expressing porcine claudin-1 exhibited a 1.5-fold and 2-fold increase, respectively, in membrane-localized claudin-1 (Fig. 3F and Fig. S2B). Importantly, claudin-1 overexpression did not affect APN expression levels (Fig. 3G). Collectively, these findings suggest that claudin-1 plays a functional role in the TGEV life cycle.

### CRISPR/Cas9 gene editing confirms claudin-1 as a TGEV host factor

**Fig 4:**
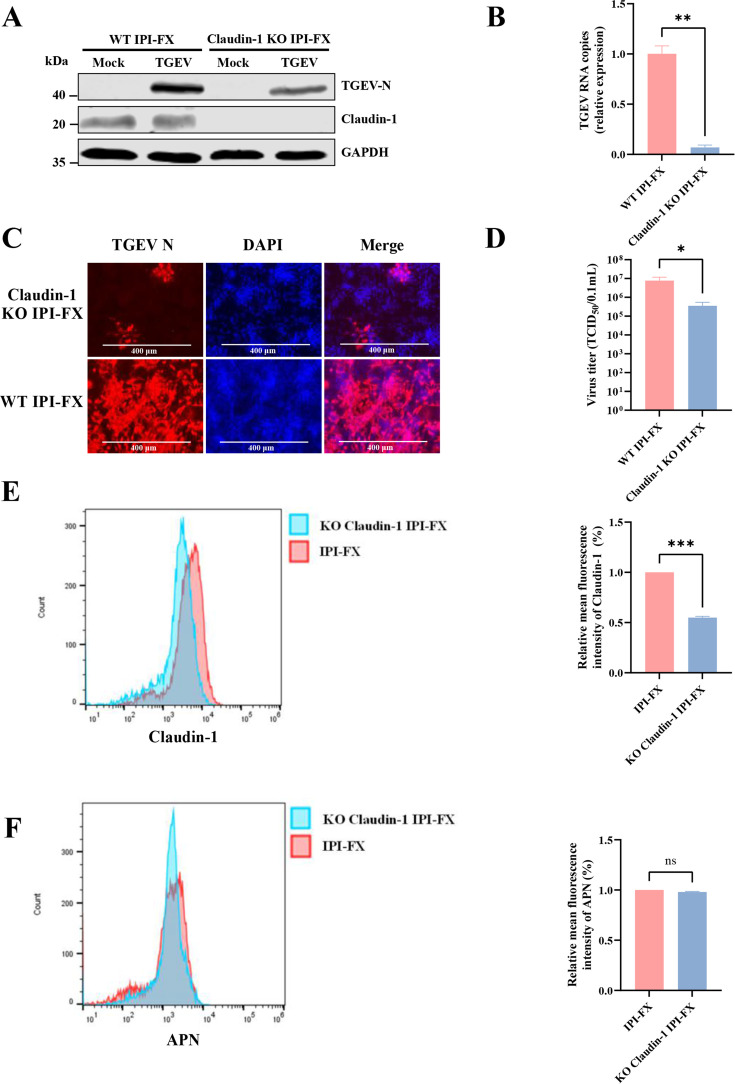
Knockout of claudin-1 inhibits the replication of TGEV. (**A**) *Claudin-1*^−/−^ IPI-FX cells and wild-type IPI-FX cells were infected with TGEV at an MOI of 0.1. The protein levels of claudin-1 and TGEV N were detected by western blotting. (**B**) The TGEV RNA copy number was detected by RT-qPCR. (**C**) At 24 hpi, the cell monolayers were fixed, and the numbers of TGEV-positive cells were determined by the use of anti-TGEV N mAb. (**D**) At 24 hpi, virus yields were determined by TCID_50_ assay with ST cells. (**E**) Under unpermeabilized conditions, the MFI of claudin-1 on the *Claudin-1*^−/−^ IPI-FX cell surface was detected by FC500 flow cytometer. (**F**) Under unpermeabilized conditions, the MFI of APN on the *Claudin-1*^−/−^ IPI-FX cell surface was detected by FC500 flow cytometer. The error bars show the standard deviations from three experiments. Significant differences: ***P < 0.001, **P < 0.01, *P < 0.05, ns, not significant.

We next used CRISPR-Cas9 to generate clonal *claudin-1* KO IPI-FX cells. Immunoblotting for endogenous claudin-1 revealed a loss in claudin-1 protein levels (Fig. 4A). These cell clones were used to further examine the importance of claudin-1 during TGEV infection. *Claudin-1* KO cells showed a 95% decrease in TGEV infection compared to the non-targeting control, confirming claudin-1 as a TGEV host factor (Fig. 4B). As shown in Fig. 4C, the susceptibility of *claudin-1* KO IPI-FX cells to TGEV infection was approximately 80% lower than that of wild-type IPI-FX cells. The reduction in the titer of progeny virus in *claudin-1* KO IPI-FX cells was confirmed by measuring the TCID_50_ (Fig. 4D). To determine whether *claudin-1* knockout affects the localization of the known receptor APN, we quantified APN membrane levels by flow cytometry under non-permeabilized conditions. The results showed that *claudin-1* knockout decreased cell surface levels of claudin-1, as expected, but did not affect the surface expression of APN (Fig. 4E and F).

To confirm the specificity of the observed phenotype and rule out off-target effects, a rescue experiment was performed in the *claudin-1* KO IPI-FX cells. Ectopic expression of claudin-1 in the KO cells significantly restored TGEV replication, as evidenced by the increased levels of TGEV-N protein and viral titers (Fig. S4). This rescue effect confirms claudin-1 as a critical host factor for TGEV infection.

### Claudin-1 is a cofactor for TGEV internalization

**Fig 5:**
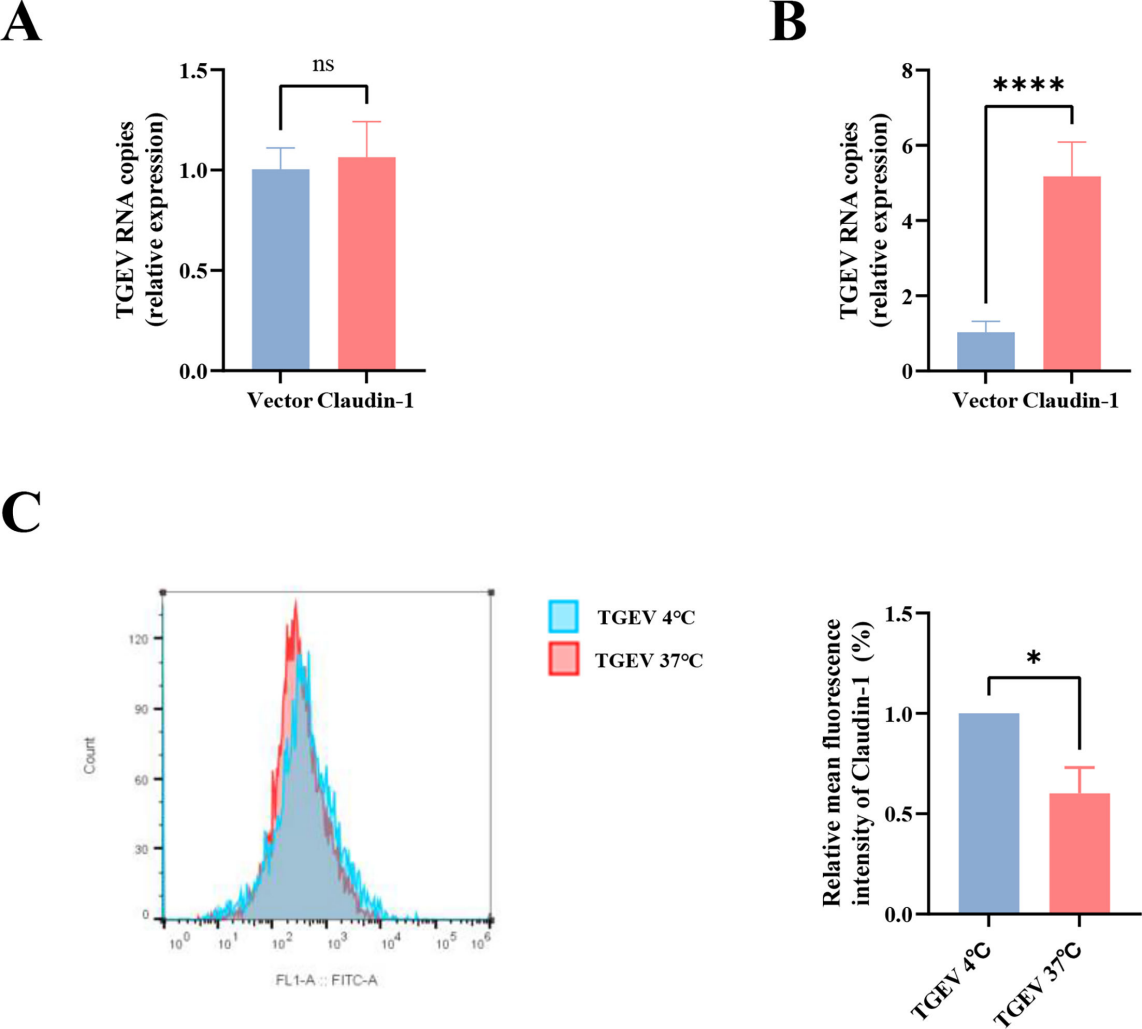
Claudin-1 is required for internalization of TGEV but not for cell binding. (**A and B**) TGEV binding (**A**) and internalization (**B**) assays were performed in IPI-FX cells by RT-qPCR. (**C**) Under unpermeabilized conditions, the MFI of claudin-1 on the cell surface was detected by FC500 flow cytometer after TGEV infection. All tests were carried out in triplicate. The error bars show the standard deviations from three experiments. ****P < 0.0001, *P < 0.05; ns, not significant.

Coronavirus infection is initiated by the binding of viral particles to specific proteins on the cell surface (36). The results described above showed that claudin-1 can promote the replication of TGEV, so we carried out TGEV virion binding assays in order to determine whether the claudin-1 protein was involved in the process of initial virus binding events. IPI-FX cells were first transfected with Myc-Claudin-1 or vector to overexpress endogenous claudin-1. At 24 h post-transfection (hpt), cells were placed at 4°C for 1 h to cool down, then incubated with TGEV for 1 h at 4°C so that virion binding, but not entry, could occur. After removal of excess virions by PBS, total RNA was extracted to determine viral levels by qPCR. As shown in Fig. 5A, there was no significant difference in TGEV attachment between control vector and claudin-1 overexpressed IPI-FX cells. Furthermore, the cells were shifted to 37°C for 1 h to allow the internalization of bound viruses. After removal of excess virions by acid buffer, cells were lysed for qPCR detection of TGEV that had entered the cells. We found that significantly more viruses entered the claudin-1 overexpressed cells than the control cells (Fig. 5B), indicating that claudin-1 affected the internalization of TGEV while not being involved in the initial attachment of virions to target cells.

We next investigated whether claudin-1 is internalized with TGEV. The expression of claudin-1 on the cell surface, both pre- and post-infection with TGEV, was quantitatively determined by using flow cytometry in non-permeabilized conditions. Notably, our results indicated a marked reduction in the cell surface expression of claudin-1 (Fig. 5C) subsequent to TGEV infection, strongly suggesting that claudin-1 undergoes internalization in response to the viral invasion.

While APN has been characterized as a receptor for TGEV, we hypothesized that it might cooperate with claudin-1 to facilitate viral internalization. To test this, we performed co-immunoprecipitation assays in HEK293T cells co-expressing Myc-tagged claudin-1 and Flag-tagged APN. The results confirmed a specific interaction between claudin-1 and APN (Fig. S3). Taken together, these findings support the role of claudin-1 as a host factor for TGEV and suggest its functional cooperation with APN during infection.

### Claudin-1 interacts with the RBD region of TGEV S protein

**Fig 6:**
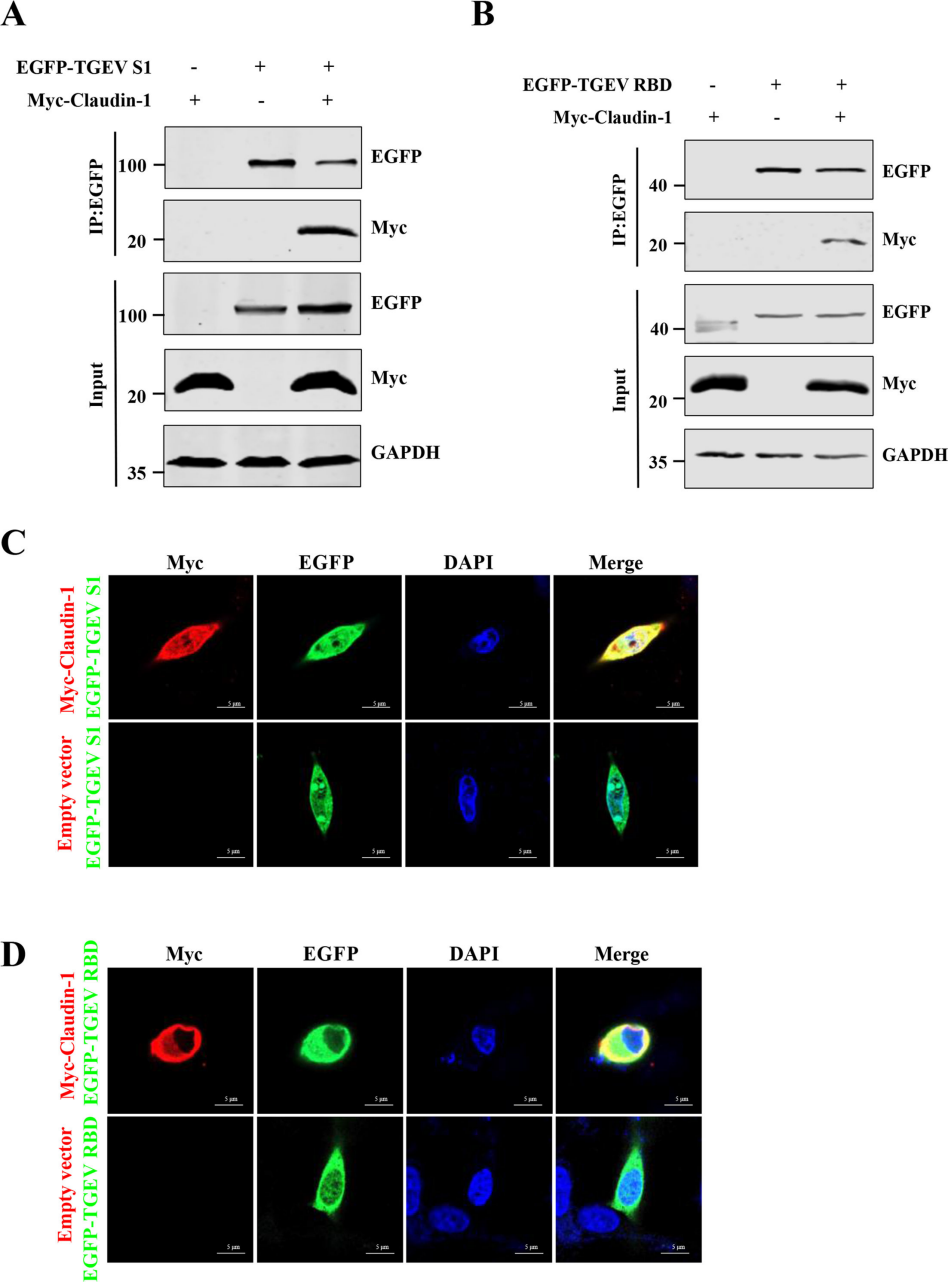
Claudin-1 exhibits colocalization and interacts with the RBD of the TGEV S protein. (**A and B**) HEK293T cells were transfected with Myc-Claudin-1 along with the EGFP-TGEV S1 or EGFP-TGEV RBD for 24 h, and then co-immunoprecipitation assays were performed with anti-GFP mAb. (**C and D**) IPI-FX cells were cotransfected with plasmids encoding Myc-Claudin-1 and EGFP-TGEV S1 or EGFP-TGEV RBD for 24 h. The cells were incubated with the rabbit anti-Myc mAb, and then the cell nuclei were stained with DAPI. Bars, 5 μm.

The first step in coronavirus infection is binding to cellular receptors via the receptor-binding domain (RBD) in S1 of the viral envelope S protein (37). We next used co-immunoprecipitation assays to test whether claudin-1 interacts with the TGEV S1 protein. Myc-tagged Claudin-1 (Myc-Claudin-1) was co-expressed with EGFP-tagged S1 protein (EGFP-TGEV S1) in HEK293T cells. Immunoblotting for EGFP-TGEV S1 demonstrated that claudin-1 interacts with the TGEV S1 protein specifically (Fig. 6A). To characterize the domain of the TGEV S1 protein that interacts with claudin-1, we performed a co-immunoprecipitation analysis using the RBD of the TGEV S protein in plasmid-transfected HEK293T cells. We found that the RBD region interacted with claudin-1 (Fig. 6B). Then, confocal microscopy revealed colocalization between claudin-1 and S1 or RBD in cells (Fig. 6C and D).

### Claudin-1 interacts with the S1 of other coronaviruses

**Fig 7:**
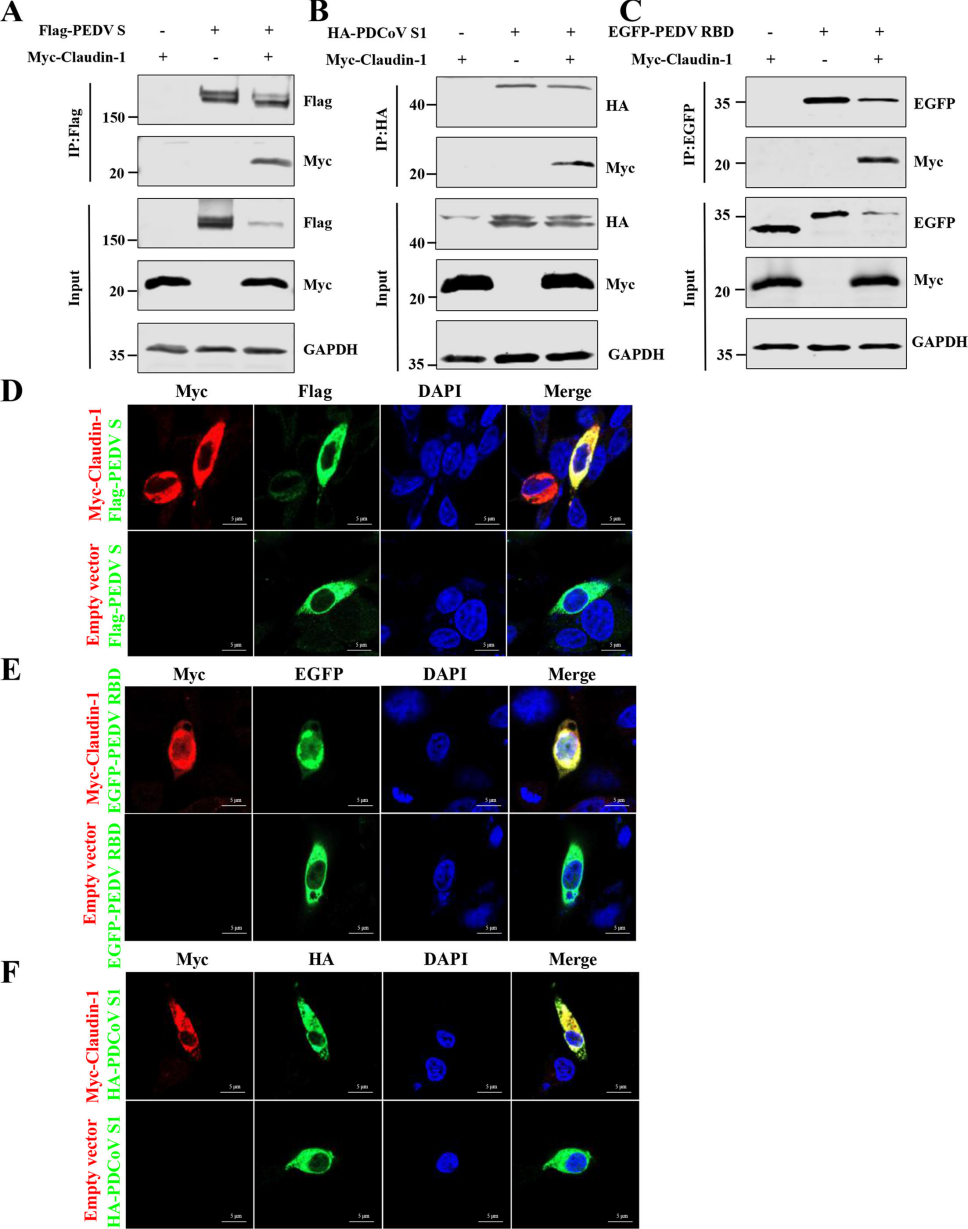
Claudin-1 exhibits colocalization and interacts with the S or S1 of PEDV and PDCoV. (**A–C**) Myc-Claudin-1 and Flag-PEDV S (**A**), HA-PDCoV S1 (**B**), EGFP-PEDV RBD (**C**) were co-transfected into HEK293T cells and then immunoprecipitated with anti-Flag, anti-HA, and anti-GFP beads. (**D–F**) IPI-FX cells were cotransfected with the Flag-PEDV S (**D**), EGFP-PEDV RBD (**E**), HA-PDCoV S1 (**F**), and Myc-Claudin-1. Fluorescence images were acquired with confocal microscopy. Bars, 5 μm.

Subsequently, we examined whether claudin-1 also interacts with the S protein of other coronaviruses. Co-immunoprecipitation assays were used to test whether claudin-1 interacts with the S or S1 protein of PEDV or PDCoV. Myc-Claudin-1 and Flag-tagged PEDV S (Flag-PEDV S) or HA-tagged PDCoV S1 (HA-PDCoV S1) were co-transfected in HEK293T cells for 24 h. The results of the co-immunoprecipitation assays indicated that claudin-1 showed an interaction with PEDV S protein and PDCoV S1 protein (Fig. 7A and B). To characterize the domain of the PEDV S protein that interacts with claudin-1, we performed a co-immunoprecipitation analysis using the RBD of the PEDV S protein in plasmid-transfected HEK293T cells. We found that the RBD of PEDV interacted with claudin-1 (Fig. 7C). Accordingly, confocal microscopy revealed colocalization between claudin-1 and PEDV S, the RBD of PEDV and PDCoV S1 in cells (Fig. 7D through F).

To investigate whether claudin-1 interacts with other coronaviruses except for SeCoVs, such as SARS-CoV-2. Myc-Claudin-1 was co-expressed with Flag-tagged SARS-CoV-2 S1 protein (Flag-SARS-CoV-2 S1) in plasmid-transfected HEK293T cells. Immunoblotting for Myc-Claudin-1 demonstrated that claudin-1 interacts with the SARS-CoV-2 S1 protein specifically (Fig. S5A). Accordingly, confocal microscopy revealed colocalization between claudin-1 and SARS-CoV-2 S1 (Fig. S5B). The results demonstrated that claudin-1 also interacts with the S1 subdomain of SARS-CoV-2.

### Claudin-1 is required for PEDV and PDCoV infection

**Fig 8:**
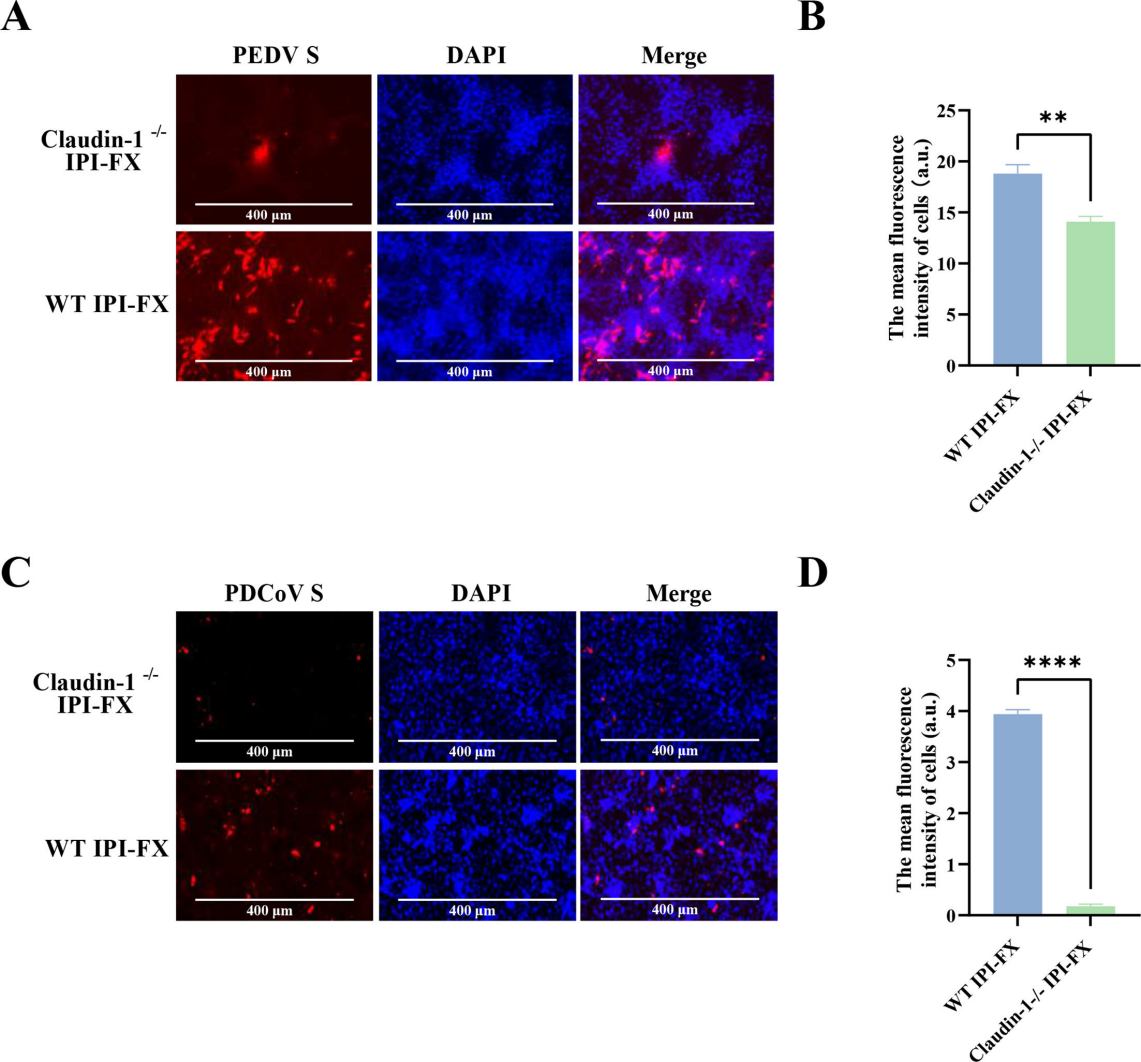
Poor replication of PEDV and PDCoV in claudin-1 knockout cell lines. (**A and C**) Wild-type IPI-FX cells and *claudin-1*^−/−^ IPI-FX cells were infected with PEDV and PDCoV (MOI = 0.1) for 24 h, and the virus infection was analyzed by IFA. (**B and D**) Image processing and analyses were conducted with ImageJ to calculate the MFI of PEDV and PDCoV-infected cells. All tests were carried out in triplicate. The error bars show the standard deviations from three experiments. Significant differences: ****P < 0.0001, **P < 0.01.

TGEV belongs to the *Coronaviridae*, we next explored whether claudin-1 could facilitate other coronaviruses' infection. To this end, we tested PEDV and PDCoV for their requirement for claudin-1. *Claudin-1*^−/−^ cells were infected with PEDV and PDCoV for 24 h. IFA assays showed that claudin-1 also affected susceptibility to PEDV and PDCoV (Fig. 8A and C). The fluorescence intensity of each cell was then calculated. We found that the fluorescence intensity in PEDV and PDCoV-infected *claudin-1*^−/−^ cells was significantly lower than that in wild-type IPI-FX cells (Fig. 8B and D). Together, these results suggest that claudin-1 is an important host factor for coronavirus infection.

### Lubiprostone induces claudin-1 and prompts PDCoV infection in piglets

**Fig 9:**
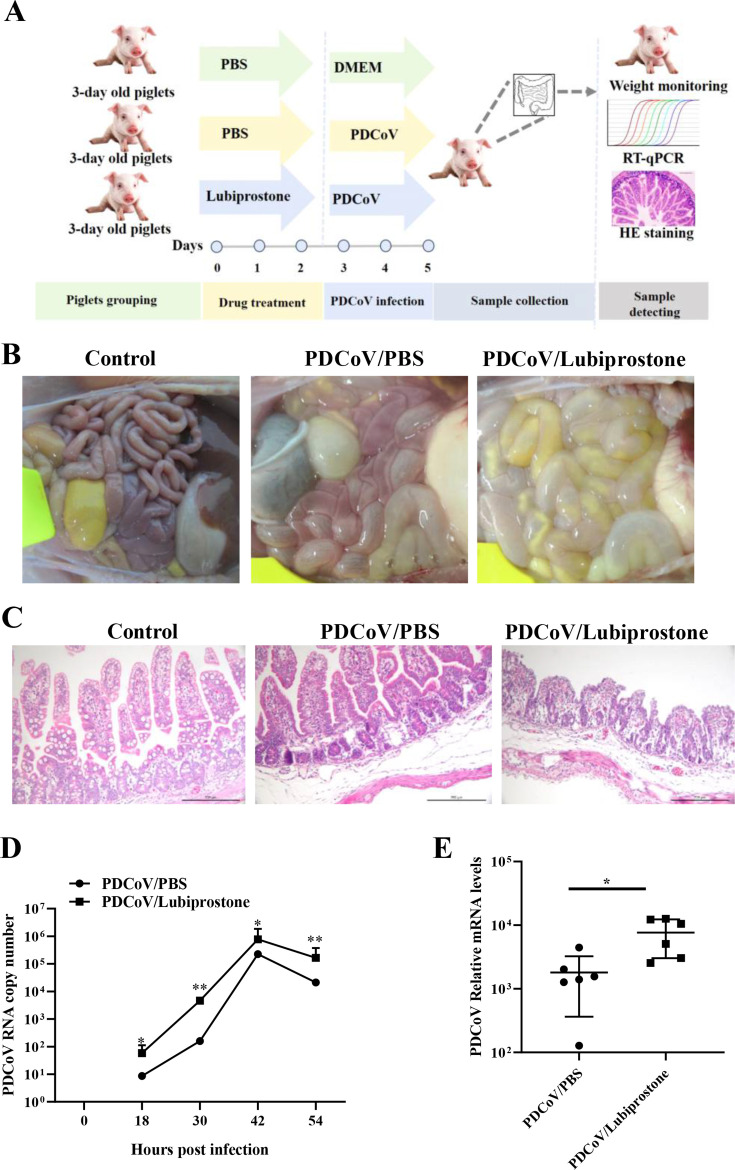
Lubiprostone induces claudin-1 and prompts PDCoV replication in piglets. (**A**) Schematic of the lubiprostone treatment and the virus challenge scheme in piglets. (**B**) The anatomical observation on the intestine of piglets. (**C**) Representative hematoxylin and eosin staining of the intestinal tissue sections of piglets. (**D**) Virus shedding of anal swabs was collected daily from the piglets and tested by RT-qPCR. (**E**) The replication of PDCoV in the intestine of piglets was detected by RT-qPCR. Significant differences: *P < 0.05.

To further verify whether claudin-1 is important for coronavirus infection *in vivo*, we used piglets to evaluate the impact of claudin-1 on PDCoV infection. Lubiprostone, a chloride channel activator, is reported to induce claudin-1 specifically (38). Here, lubiprostone (10 μg/kg) was administered orally twice a day before PDCoV infection (Fig. 9A). At 20 h after challenge, all piglets in the PDCoV/lubiprostone group exhibited severe watery diarrhea of PDCoV infection, which appeared 10 h earlier than in the PDCoV/PBS group. One piglet in the PDCoV/lubiprostone group was on the brink of death 54 h after infection and was euthanized shortly after. The remaining piglets were euthanized 72 h after PDCoV infection. Anatomical observations showed that piglets infected with PDCoV exhibited typical lesions in the intestine, including thinning of the intestinal wall and watery intestinal contents. The PDCoV/lubiprostone group piglets exhibited intestinal dilation with an abundance of yellow liquid (Fig. 9B).

Histopathological examination of the intestine revealed typical features of viral enteritis in the PDCoV/PBS group, including shortening and shrinking of villi in the small intestine and mild edema in the intestinal submucosa. The PDCoV/lubiprostone group showed worse damage to the small intestine, characterized by villous atrophy, intestinal mucosal epithelial necrosis, and shedding (Fig. 9C).

To assess whether claudin-1 expression is modulated by lubiprostone treatment, we measured its levels in both control and PDCoV/lubiprostone groups. As shown in Fig. S6, claudin-1 expression was significantly upregulated in the lubiprostone-treated group. Since lubiprostone is known to selectively stimulate type 2 chloride channels and promote chloride efflux into the intestinal lumen, we further evaluated whether chloride channels were involved in this process. Specifically, we examined the expression of chloride intracellular channel proteins CLIC1 and CLIC4 in the same samples and found no significant changes in their protein levels between the control and PDCoV/lubiprostone groups (Fig. S6A). Immunohistochemistry was used to detect the expression level of claudin-1 in the jejunum, and the results showed that lubiprostone treatment increases claudin-1 levels within the PDCoV-infected intestine epithelial cells (Fig. S6B).

Anal swabs were collected daily from the piglets to detect and quantify any shedding of the virus. The results showed that the viral load in fecal swabs of the PDCoV/lubiprostone group was higher than that of the PDCoV group (Fig. 9D). Assessment of viral replication in the intestine by qPCR demonstrated PDCoV/lubiprostone piglets to have significantly increased viral RNA copies in their jejunum and ileum, when compared to the PDCoV/PBS group piglets (Fig. 9E). Overall, lubiprostone treatment prompts PDCoV infection in the piglets.

## DISCUSSION

Tight junction proteins play a pivotal role in maintaining the structural integrity and functional selectivity of epithelial and endothelial barriers (39). These proteins form a continuous seal between adjacent cells, creating a selectively permeable barrier that regulates the passage of solutes, ions, water, and even larger particles, such as pathogens and inflammatory molecules, between the cells and the extracellular environment (40). Claudin-1 belongs to the claudins subfamily, which comprises a diverse group of transmembrane proteins that contribute significantly to the formation and regulation of tight junctions (41). Claudin-1 is one of the most abundant claudins expressed in various tissues, particularly in the epithelia of the skin, kidney, and gut (42). It is worth noting that, in this study, the expression of *ZO-1*, *occludin*, and *Muc2* in the intestinal tissue was significantly decreased, while the expression of *claudin-1* in the jejunum was significantly higher in TGEV-infected piglets than that in the normal animals. Similar to that of TGEV, PEDV, and PDCoV infection increased the expression of claudin-1 in the jejunum and ileum of weaned piglets (23, 43). These results emphasize the potential importance of claudin-1 in SeCoV infection. Here, knockout of *claudin-1* in TGEV-permissive IPI-FX cells significantly reduces virus infection, especially during the internalization stage and subsequent viral replication. In contrast, overexpression of claudin-1 significantly enhances both virus internalization and replication but not virus binding. The discovery that claudin-1, a crucial member of the tight junction protein family, functions as an internalization factor for coronaviruses, including TGEV, PEDV, and PDCoV, represents a significant breakthrough in understanding the viral infection mechanism. This finding not only sheds new light on the complex interactions between host proteins and coronaviruses but also provides potential targets for the development of novel prevention and control strategies.

The therapeutic potential of claudin-1 has been increasingly explored in cancer research. Using a novel monoclonal antibody (6F6) that specifically targets the extracellular domain of human claudin-1, researchers have demonstrated that claudin-1 inhibition reduces the growth of colorectal cancer xenografts and suppresses the formation of liver metastases (44). Regulation of claudin protein expression may help control tumor invasiveness. Clinical studies in this area are primarily focused on combining claudin-targeted therapies with conventional treatments. For example, anti-claudin-5 antibodies have been investigated in combination with small-molecule inhibitors and chemotherapeutic agents, such as paclitaxel and carboplatin (45). Furthermore, given that claudin-1 serves as a co-receptor for HCV (46), and as shown in this study, for enteric coronaviruses, these inhibitors may pave the way for novel therapeutic strategies.

TGEV Miller exhibited a higher infectivity in APN-positive cells than in APN-negative cells. In contrast, TGEV Purdue replicated more efficiently in APN-negative cells. PEDV infected both APN-positive and negative enterocytes (47). Porcine alveolar macrophages (PAMs) from *ANPEP* KO pigs exhibited resistance to PDCoV infection. However, lung fibroblast-like cells, derived from the *ANPEP* KO PAM cultures, supported high levels of PDCoV infection (14). These results showed that an additional receptor exists, distinct from APN for porcine coronaviruses. The efficient initiation of viral infection often necessitates more than just binding to a single cell surface protein, with viruses frequently relying on one or more coreceptors, dependent upon the target cell type and entry steps (48). Multiple host factors have emerged as potential coreceptors facilitating coronavirus infections. Notably, a study utilizing genomic receptor profiling revealed the ACE2/ASGR1/KREMEN1 (ASK) receptor combination employed by SARS-CoV-2 across various cell types (49). Tight junction protein occludin is an internalization factor for SARS-CoV-2 and PEDV infection (24, 50). Claudin-1, identified in this study, has been identified as a coreceptor for HCV (30). Here, we reveal a novel role of claudin-1 as an internalization factor for TGEV, PEDV, and PDCoV entry and further demonstrate that SARS-CoV-2 S1 binds to claudin-1 (Fig. S5). Whether claudin-1 is an internalized receptor of SARS-CoV-2 deserves further study.

Our findings demonstrate that TGEV infection significantly upregulates the tight junction protein claudin-1 in intestinal epithelial cells (Fig. 2). Interestingly, this upregulation was observed not only in virus-infected (antigen-positive) cells but also prominently in uninfected cells. We hypothesize that this phenomenon in uninfected cells is mediated by paracrine signaling from infected neighbors. Since TGEV-infected cells can activate MAPK signaling known to regulate tight junction proteins (51–53). A plausible explanation is that MAPK signaling may initiate the release of soluble factors that diffuse to neighboring uninfected cells, inducing claudin-1 expression. The resultant increase in claudin-1 in bystander cells markedly enhances their susceptibility to infection, effectively “priming” the surrounding tissue. This priming enables newly produced virions from initially infected cells to efficiently initiate a secondary infection wave, representing a highly effective strategy for viral amplification and dissemination. The underlying mechanism requires further investigation. Similar mechanisms have been reported in other viral infections, such as HCV, which also utilizes claudin-1 as a co-receptor (54). Therefore, the widespread upregulation of claudin-1 in infected and uninfected cells is not an insignificant epiphenomenon, but rather a key event that may significantly enhance the dissemination efficiency and pathogenicity of SeCoV within the epithelial tissue.

To determine whether claudin-1 is important for coronavirus infection *in vivo*, lubiprostone treatment was used to induce the expression of claudin-1. Lubiprostone was safe, well-tolerated, and associated with limited adverse effects (55, 56). One study has demonstrated the claudin-1 induction potential of lubiprostone; however, this finding has been primarily limited to *in vitro* characterizations (38). Here, lubiprostone treatment increases claudin-1 levels within the PDCoV-infected intestine cells (Fig. S6). Through pharmacological induction of claudin-1, we demonstrate the induction effect in piglets, and this activity is important for PDCoV replication in intestinal epithelial cells. Collectively, this study demonstrates claudin-1 as an internalization factor for TGEV, PEDV, and PDCoV. We also highlight that claudin-1 directly binds to SARS-CoV-2 S1. The results reveal important mechanistic details of the role of claudin-1 in the internalization step of the coronavirus entry.

## MATERIALS AND METHODS

### Cells, viruses, and antibodies

IPI-FX cells were derived from IPI-2I cells (porcine ileum epithelial cells). Swine testicular (ST) cells, African green monkey kidney cells (Vero E6) cells, and human embryonic kidney 293T (HEK293T) cells were frozen and stored by our laboratory and cultured in Dulbecco′s modified Eagle′s medium (Thermo Fisher, Waltham, MA, USA, Catalog #C11995500BT) supplemented with 10% fetal bovine serum (Thermo Fisher, Catalog #10270106). All cells were cultured in a humidified atmosphere at 37°C and 5% CO_2_. The TGEV strain H165 (GenBank accession no. EU074218) and the PDCoV strain NH (GenBank accession no. KU981062.1) used in this study were propagated in ST cells and preserved in our laboratory (57). PEDV strain CV777 (GenBank accession no. KT323979) was grown and titrated in Vero E6 cells.

Antibodies were obtained from the following sources. Anti-GAPDH (Sigma-Aldrich, St. Louis, MO, USA, Catalog #ABS16), anti-Flag (Sigma-Aldrich, Catalog #F1804), anti-HA (Sigma-Aldrich, Catalog #H6908), and anti-Myc (Sigma-Aldrich, Catalog #M4439) were from Sigma-Aldrich. Anti-claudin-1 was purchased from Abmart (Shanghai, China, Catalog #T56872F) and Santa Cruz Biotechnology (Santa Cruz, CA, USA, Catalog #sc-166338). Anti-GFP was purchased from Proteintech (Chicago, Illinois, USA, Catalog #50430-2-AP). The mAbs against TGEV N, PDCoV S, and PEDV S were made in our laboratory.

### Plasmid construction and transient transfection

The S gene of PEDV CV777 (GenBank accession no. JN599150.1) was amplified from PEDV-infected IPI-FX cells by RT-PCR and cloned into p3×FLAG-CMV-10. The S1 gene of SARS-CoV-2 was amplified from pcDNA3.1-SARS-CoV-2-Spike-Myc (Beyotime, Shanghai, China, Catalog #D2945) and cloned into p3×FLAG-CMV-10. The S1 of TGEV was amplified and cloned into the pEGFP-C2 vector, named EGFP-TGEV S1. The RBD region of TGEV and PEDV was amplified and cloned into the pEGFP-C2 vector, named EGFP-TGEV RBD and EGFP-PEDV RBD. The amplified claudin-1 was conjugated to the pCMV vector with a C-terminal Myc tag. The constructed S1 of PDCoV with a C-terminal HA tag in the pCAGGS vector was stocked in our laboratory. All constructs were verified by DNA sequencing. The primers are listed in Table 1. IPI-FX cells were seeded onto 6-well plates at a density of 2.5 × 10⁵ cells per well in complete growth medium. Subsequently, 1.5, 2, and 2.5 µg of plasmid were diluted in 250 µL of serum-free medium. IPI-FX cells were transfected with plasmids with Lipofectamine 3000 (Invitrogen, Waltham, MA, USA, Catalog #L3000015), according to the manufacturer′s instructions. HEK293T cells were transfected with X-tremeGENE HP DNA transfection reagent (Roche, Basel, Switzerland, Catalog #6366236001) according to the manufacturer′s instructions.

**TABLE 1:** Sequences of primers used to construct plasmids

Vector	Primer	Sequence (5′−3′)
pEGFP-C2	TGEV S1-F	GGCCGGACTCAGATCTACATGAAGAAGCTGTTCGTGGTGC
	TGEV S1-R	GATCCCGGGCCCGCGGTACCTTACACGCCGTCGGACACGT
	TGEV RBD-F	GGCCGGACTCAGATCTACATGGTGAACAACATCAAGTGCAGC
	TGEV RBD-R	GATCCCGGGCCCGCGGTACCTCACACTCCCACGATATTGTCGC
	PEDV RBD-F	GGCCGGACTCAGATCTACATGGAGTCTTTAACCTACTTCTGGTTGTT
	PEDV RBD-R	GATCCCGGGCCCGCGGTACCTCAGCCATAACCACCTCTAATATGGCT
pCMV-Myc	Claudin-1-F	CGGTCGACCGAGATCTCGATGGCCAACGCGGGG
	Claudin-1-R	ATCCCCGCGGCCGCGGTACCTCACACGTAGTCTTTCCCACTGGAAG
p3×FLAG-CMV-10	PEDV S-F	TTGCGGCCGCGAATTCAATGAGGTCTTTAATTTACTTCTGGTTGCTCT
	PEDV S-R	CTCTAGAGTCGACTGGTACCTCACTGCACGTGGACCTTTTCAAA
	SARS-CoV-2 S1-F	TTGCGGCCGCGAATTCAATGTTCGTGTTCCTCGTGCTG
	SARS-CoV-2 S1-R	ATGCCACCCGGGATCCTCAAGCTGGGGCCACCTG

### CRISPR/Cas9-mediated knockout of *claudin-1* in IPI-FX cells

Using CRISPR-Cas9 technology, *claudin-1* gene knockout (KO) cells were produced in IPI-FX cells. To achieve this, we first utilized the CRISPR design website (https://portals.broadinstitute.org/gpp/public/analysis-tools/sgrna-design) to select the sgRNA targeting regions. The sgRNA sequences were 5′-CACCGGGCCCAGGCCATCTACGAG-3′, 5′-CACCGCCCGTGCCTTGATGGTAAT-3′, and 5′-CACCGCTATCTTAGTTGCCACAGCA-3′. The primers were then synthesized by Comate Bioscience Company (Jilin, China). Following the annealing of the target sequence oligonucleotide pairs, the resulting fragments were cloned into the pX330-U6-Chimeric_BB-CBh-hSpCas9 vector (plasmid # 42,230 from Feng Zhang, obtained from Addgene) and subsequently co-transfected with pEGFP-C2 into IPI-FX cells. At 24 hpt, EGFP-positive cells were sorted into single clones in the 96-well plates by flow cytometry using the SONY-MA900 Flow Cell Sorter. Individual cell colonies were collected by trypsinization and cultured in 96/48-well plates and sub-cultured in 12-well plates. Western blotting and Sanger sequencing were used to validate the knockout cell clones. Finally, the *claudin-1*-KO clone used in the following study was derived from sgRNA: 5′-CACCGGGCCCAGGCCATCTACGAG-3′.

### Establishment of IPI-FX stably expressing porcine claudin-1

To validate the functionality of claudin-1, we generated claudin-1-expressing lentiviruses by co-transfecting a bicistronic lentiviral vector encoding claudin-1 fused with a C-terminal HA tag and ZsGreen (green fluorescent protein) along with the packaging plasmids psPAX2 and pMD2.G into HEK293T cells, utilizing the X-tremeGENE HP DNA transfection reagent from Roche. The primers utilized for cloning are presented in Table 2. The supernatant containing the claudin-1-expressing lentiviruses was then employed to infect IPI-FX cells, and polybrene was added at a ratio of 1:1,000. Then the infected cells were maintained under selective pressure with 2 µg/mL of puromycin, replacing the media every 3–4 days.

**TABLE 2:** Sequences of primers used to construct plasmids

Vector	Primer	Sequence (5′−3′)
pLVX-IRES-ZsGreen1	HA-Claudin-1-F	CGGTGAATTCCTCGAGAGATGTACCCATACGATGTTCCAGATTACGCTGCCAACGCGGGGCTGCAGCTGC
	HA-Claudin-1-R	GAGAGGGGCGGGATCCTCACACGTAGTCTTTCCCACTGGAAG

### Reverse transcription-quantitative polymerase chain reaction

Following the manufacturer’s instructions, RNA was extracted from 24-well plates using the Simply P total RNA extraction kit (BioFlux, Beijing, China, Catalog #BSC52S1). To reverse transcribe total RNA into complementary DNA (cDNA), M-MLV reverse transcriptase (Takara, Otsu, Shiga, Japan, Catalog #2641A) and an Oligo(dT)_15_ primer (Takara, Catalog #3805) were utilized. Subsequently, the cDNA samples were subjected to qPCR using FastStart Essential DNA Green Master (Roche, Catalog #6402712001). The primers of *claudin-1*, *occludin*, *ZO-1*, *Muc2*, *TGEV S, PEDV N*, *PDCoV N*, and *APN* genes generated by the Oligo 6 program are displayed in Table 3. The data were analyzed using the cycle threshold (ΔΔ*C_T_*) approach and normalized to glyceraldehyde-3-phosphate dehydrogenase (GAPDH) expression.

**TABLE 3:** Sequences of RT-qPCR primers

Primer	Sequence (5′−3′)
Claudin-1-F	CCAGTGCAAAGTCTTCGACTC
Claudin-1-R	CTGCACCTCATCATCTTCCAT
Occludin-F	CAGAGCAGGAAAGTCTAGGAGA
Occludin-R	GATCTGAAGTGATGGGTGGATA
ZO-1-F	GAGGAAATGATGAGAGCCAAC
ZO-1-R	CTCATATCTGTACGCAGGCTG
Muc2-F	GTATCGACCACCACAGTGACTC
Muc2-R	GTCCTCAAGGGATTCAAAGTCT
TGEV S-F	GCTTGATGAATTGAGTGCTGATG
TGEV S-R	CCTAACCTCGGCTTGTCTGG
PEDV N-F	GCAGTAATTCCTCAGATCCTC
PEDV N-R	GTAGTGTCAGATGCAATGAG
PDCoV N-F	AGCAACCACTCGTGTTACTTG
PDCoV N-R	CAACTCTGAAACCTTGAGCTG
APN-F	TGTTTGACCCACAGTCCT
APN-R	TCCACATAGGAGGCAAAG
GAPDH-F	CCTTCCGTGTCCCTACTGCCAAC
GAPDH-R	GACGCCTGCTTCACCACCTTCT

### Virus titration

ST cells were seeded in a 96-well plate at 37°C and 5% CO_2_ for 24 h, and were infected with a gradient 10-fold dilution of virus supernatants. Eight replicate wells were set for each viral gradient dilution. The cells were incubated for 3 to 5 days. Based on cells showing the expected cytopathic effects, TCID_50_ was calculated using the Reed-Muench method.

### Viral binding assay

The quantification of cell-bound TGEV was conducted via RT-qPCR. Prior to inoculation, cells were transfected with Myc-Claudin-1 and incubated for 24 h. Subsequently, the cells were chilled on ice for 30 min and inoculated with TGEV at an MOI of 50. This step was followed by an hour-long incubation at 4°C. Subsequently, unbound virions were eliminated through three washes with pre-chilled PBS, and the cells were lysed using the Simply P total RNA extraction kit (BioFlux, Catalog #BSC52M1). Finally, the viral RNA expression level relative to GAPDH was determined using qPCR.

### Viral internalization assay

Internalized virions were detected by the use of RT-qPCR. Cells were transfected with Myc-Claudin-1 for 24 h and then transferred onto ice for 20 min. Cells were then infected with TGEV (MOI = 50) and placed at 4°C for 1 h. After removal of unbound virions by extensive washing with chilled PBS, the cells were moved to 37°C for 1 h to allow internalization. Upon completion of the internalization period, the cells were rigorously washed three times for 3 min with acidic buffer (50 mM glycine, 100 mM NaCl, pH 3.0). This step aimed to detach any TGEV remaining attached to the cell surface, which was subsequently removed via trypsinization. The cells were lysed for total RNA extraction and subjected to qPCR to detect internalized viruses.

### Flow cytometry

To detect claudin-1 on the cell membrane after virus infection, IPI-FX cells (6 × 10^5^ cells/well) were seeded onto 6-well plates for 24 h and then infected with TGEV (3 × 10^7^ TCID_50_/well) in a volume of 2 mL at 4°C for 1 h or at 37°C for 1 h. The cells were fixed with 4% paraformaldehyde at room temperature for 15 min, then washed three times with fluorescence-activating cell sorter wash buffer (PBS containing 2% FCS), and incubated for 2 h with claudin-1 antibody (Santa Cruz Biotechnology, Catalog #sc-166338). They were then washed and stained with goat anti-mouse Alexa Fluor 647 (Thermo Fisher, Catalog #A11003) for 1 h. All cells were subjected to flow cytometric analysis using an FC500 flow cytometer (Beckman Coulter, Fullerton, CA, USA). The MFI of claudin-1 on the cell surface was quantitatively measured and analyzed using FlowJo software (FlowJo LLC). The MFI of claudin-1 and APN was assessed as described above in claudin-1 KO IPI-FX cells, claudin-1-overexpressing cells, and IPI-FX cells stably expressing porcine claudin-1.

### Co-immunoprecipitation assay and western blotting

To detect the interaction between claudin-1 and S1 protein of TGEV, HEK293T cells grown in 6-well plates were co-transfected with 2 µg of plasmids Myc-Claudin-1 and EGFP-TGEV S1 using X-tremeGENE DNA transfection reagent. Simultaneously, cells co-transfected with Myc-Claudin-1 and pEGFP-C2, as well as pCMV-Myc and EGFP-TGEV S1, were set as control. Transfected cells were harvested at 24 hpt. After 24 hpt, the HEK293T cells were lysed in NP40 lysis buffer (Beyotime, Catalog #P0013F) containing 1% phenylmethylsulfonyl fluoride (PMSF) by incubation at 4°C for 30 min, followed by centrifugation at 12,000 × *g* at 4°C for 10 min. Supernatants were precipitated with the indicated protein mAb and incubated with gentle rocking overnight at 4°C. Protein A/G beads washed with PBS were added to supernatant fractions. After the addition, protein A/G beads incubation was continued for 12 h at 4°C on a rocker platform. The beads were washed five times with PBST and then analyzed by western blotting using mouse anti-GFP monoclonal antibody (Proteintech, Catalog #50430-2-AP). The interactions between TGEV RBD, PEDV S, PEDV RBD, PDCoV S1, SARS-CoV-2 S1, and claudin-1 were tested using the same methods with protein A/G beads and western blotting using anti-GFP, anti-Flag (Sigma-Aldrich, Catalog #F1804), and anti-HA (Sigma-Aldrich, Catalog #H6908) monoclonal antibody. For western blotting, treatedt cells were lysed in NP40 lysis buffer mixed with PMSF for western blotting. SDS-PAGE was used to separate equal quantities of extract after centrifugation, and the gels were transferred to polyvinylidene difluoride membranes (Beyotime, Catalog #FFP22). Following 5% skim milk (BD Difco, Sparks, MD, USA, Catalog #232100) blocking, the membranes were immunoblotted with the relevant primary antibody and then incubated with an appropriate IRDye-conjugated secondary antibody (Li-Cor Biosciences, Lincoln, Nebraska, USA).

### Immunofluorescence assay

IPI-FX cells were grown on coverslips in 96-well plates and were transfected with the Myc-Claudin-1 for 24 h, then infected with TGEV (MOI = 0.1) for 24 h. The cells were washed three times with PBS, and fixed in 4% paraformaldehyde for 30 min at 4°C and permeabilized with 0.2% Triton X-100 (Sigma-Aldrich, Catalog #T9284) for 20 min at room temperature. After blotting with 5% skim milk in PBS for 1 h at 37°C, the samples were then incubated at 37°C for 2 h with the primary antibody and incubated with corresponding secondary antibodies for 1 h at 37°C. After being incubated with DAPI) (Sigma-Aldrich, Catalog #S7113) for 10 min at room temperature, the cells were visualized with an EVOS FL Cell Imaging System fluorescence microscope (Thermo Fisher Scientific).

### Confocal microscopy

IPI-FX cells were grown in glass slides and cotransfected with Myc-Claudin-1, EGFP-TGEV-S1, EGFP-TGEV RBD, Flag-PEDV S, EGFP-PEDV RBD, HA-PDCoV S1, Flag-SARS-CoV-2 S1, Flag-APN, or empty vector (pCMV-Myc, pEGFP-C2, pCAGGS-HA, and p3×FLAG-CMV-10). At 24 hpt, the cells were washed three times with PBS, fixed with 0.4% paraformaldehyde for 30 min at 4°C, then washed three times with PBST. Subsequently, the cells were permeabilized with 0.2% Triton X-100 for 20 min at room temperature and blocked with 5% skim milk in PBS for 1 h at 37°C. Then incubated with the primary antibody and the corresponding secondary antibodies. The cells were stained with DAPI for 5 min at room temperature and visualized using a Zeiss LSM 980 confocal microscope (Zeiss, Oberkochen, Germany).

### Animal experiment

Nine 3-day-old piglets were divided into three groups (*n* = 3 per group): a negative control group, a PDCoV/PBS group, and a PDCoV/lubiprostone group. Following 2 days of treatment with either lubiprostone (10 μg/kg) or PBS via oral gavage twice daily, the piglets were challenged orally with 3 mL of PDCoV (5 × 10⁶ TCID₅₀). The negative control group received PBS instead of the virus. All piglets were monitored over the 3-day infection period for illness score.

### Statistical analysis

Variables are expressed as means ± standard deviations (SD). All results were analyzed using Student’s *t*-test or one-way analysis of variance, and analyzed using GraphPad Prism 9. *P* values of <0.05 were considered significant.

## SUPPLEMENTAL MATERIAL

Supplemental figures mbio.03496-25-s0001.doc Fig. S1-S6.

ASM does not own the copyrights to Supplemental Material that may be linked to, or accessed through, an article. The authors have granted ASM a non-exclusive, world-wide license to publish the Supplemental Material files. Please contact the corresponding author directly for reuse.
