# Supplementary material for: Tight junction protein claudin-1 is a novel internalization factor for swine enteric coronaviruses infection
Source: mBio. 2026 Jan 9;17(2):e03496-25. doi: 10.1128/mbio.03496-25 (PMC12892932; doi:10.1128/mbio.03496-25)
Supplement: Supplemental figures — Fig. S1-S6. [file mbio.03496-25-s0001.doc]

**Tight junction protein claudin-1 is an internalization factor for swine enteric coronaviruses infection**

Zhongyuan Li, Jianfei Chen, Yunyan Chen, Shouping Hu, Huan Li, Liang Li, Mei Xue*, Li Feng*

State Key Laboratory for Animal Disease Control and Prevention, Harbin Veterinary Research Institute, Chinese Academy of Agricultural Sciences, Harbin, Heilongjiang, China

*Corresponding Author, Mei Xue ([xuemei_23@126.com](mailto:xuemei_23@126.com)), Li Feng ([fengli@caas.cn](mailto:fengli@caas.cn))


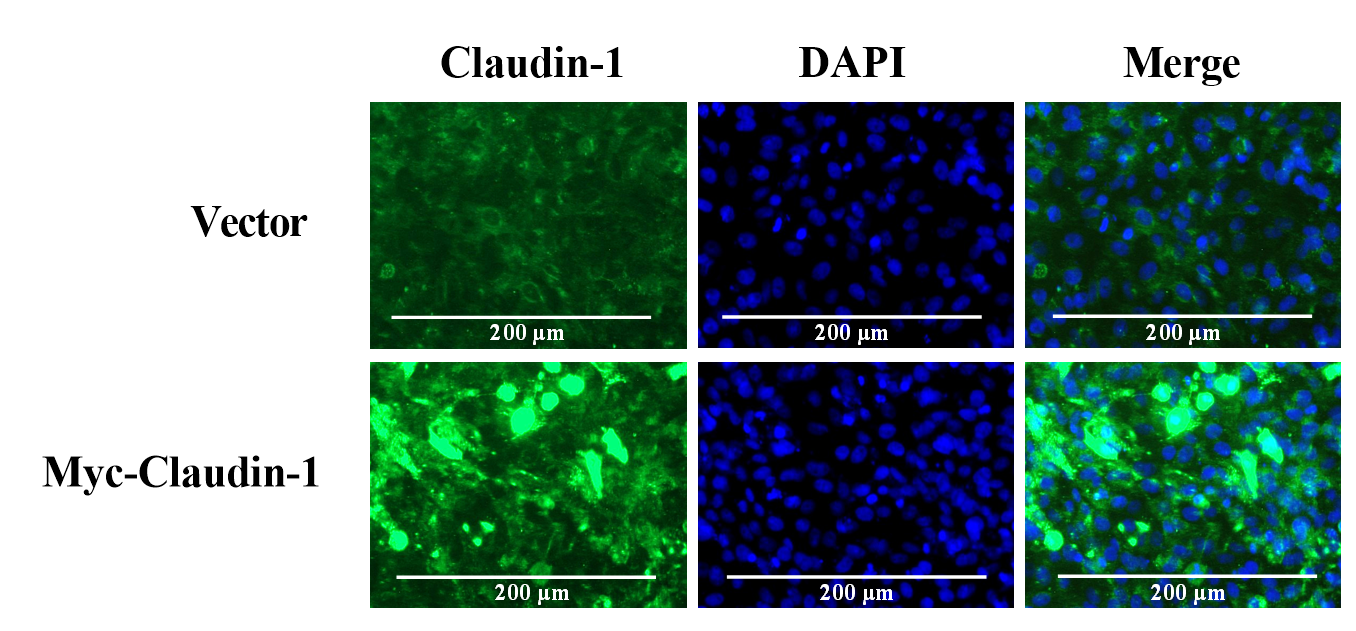


**Fig. S1. Myc-claudin-1 overexpression substantially elevated the basal expression level of endogenous claudin-1.** The basal level of endogenous claudin-1 expression in claudin-1 overexpressed cells was measured by IFA.


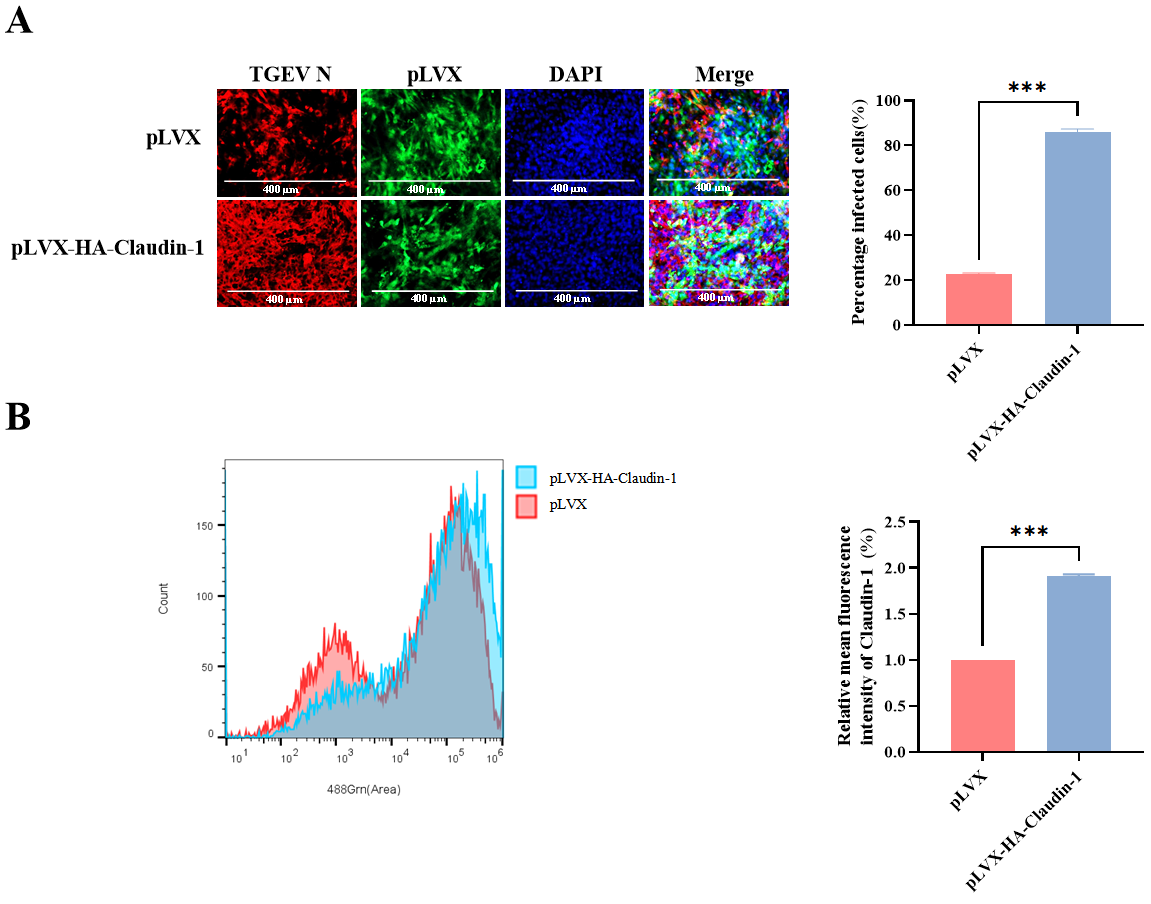


**Fig. S2. Claudin-1 facilitates the proliferation of TGEV.** (A) IPI-FX cells were transfected with the bicistronic lentiviral vector expressing porcine claudin-1 or vector and then infected with TGEV (MOI=0.1). At 24 hpi, the cells were fixed and stained with corresponding antibodies. Image processing and analyses were conducted with Image J to calculated the mean fluorescence intensity (MFI) of TGEV infected cells. (B) Under unpermeabilized conditions, the MFI of claudin-1 on the cell surface was detected by FC500 flow cytometer in IPI-FX cells stably expressing porcine claudin-1. All tests were carried out in triplicate. The error bars show the standard deviations from three experiments. Significant differences: ****P*<0.001.


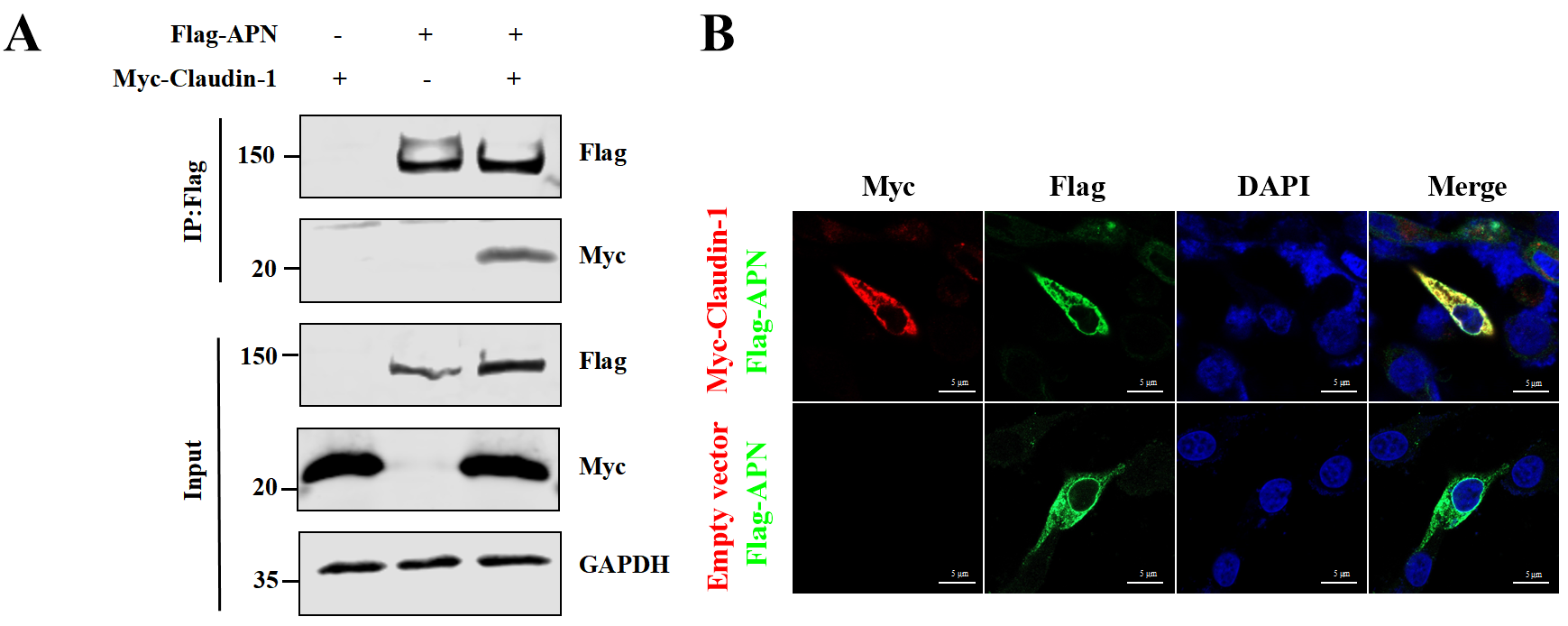


**Fig. S3. Claudin-1 exhibits colocalization and interacts with APN. (**A) HEK293T cells were co-transfected with Myc-Claudin-1 and the Flag-APN for 24 h, and then co-immunoprecipitation assays were performed with anti-Flag mAb. (B) IPI-FX cells were co-transfected with plasmids encoding Myc-Claudin-1 and Flag-APN for 24 h. Fluorescence images were acquired with confocal microscopy. Bars, 5 μm.


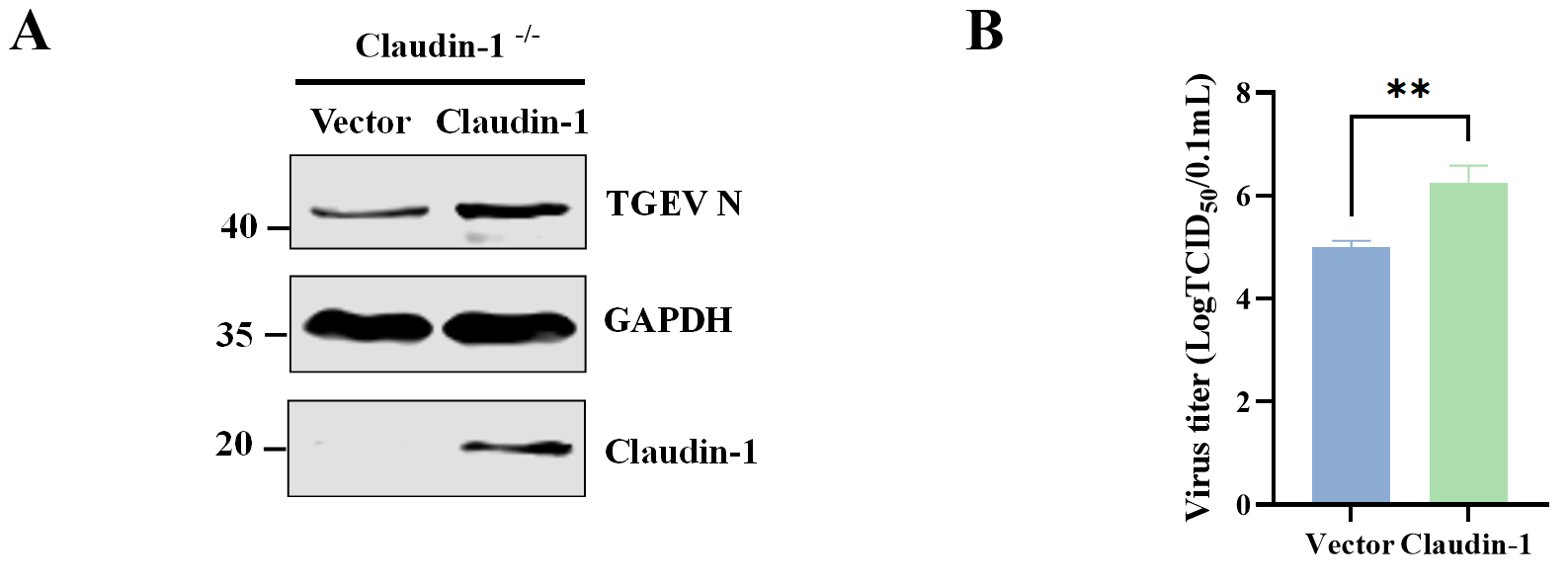


**Fig. S4. Claudin-1 reconstitution rescued the attenuated TGEV replication phenotype in *claudin-1* KO cells.** (A) *Claudin-1* KO cells were transfected with Myc-Claudin-1 plasmid or a vector control for 24 h and infected with TGEV (MOI=0.1) for 24 h. The overexpression of claudin-1 and the replication of TGEV was assayed by Western blotting. (B) At 24 hpi, virus titers were determined by TCID_50_ assay in ST cells.


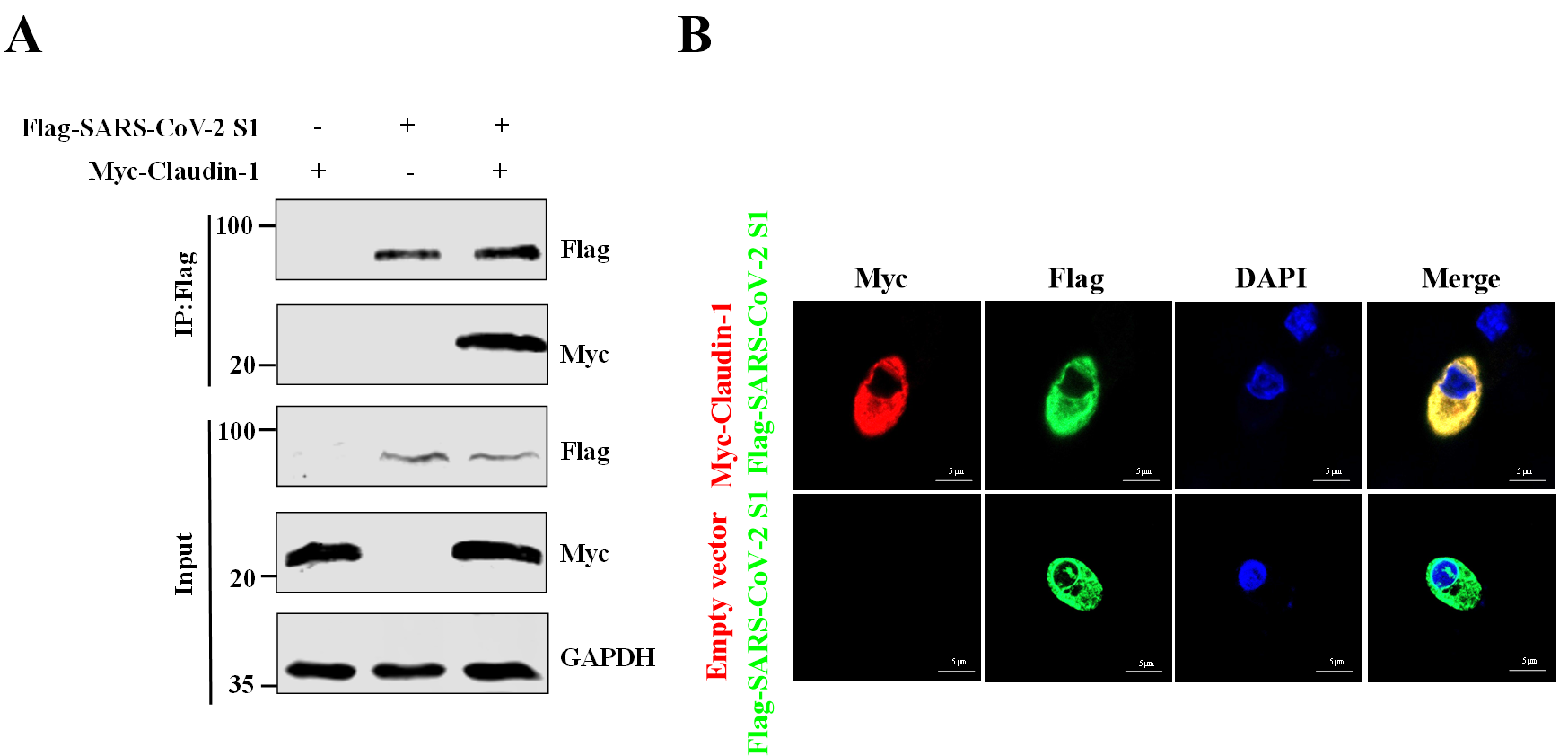


**Fig. S5**. **SARS-CoV-2 S1 interacts with claudin-1.** (A) The interaction of SARS-CoV-2 S1 and claudin-1 was detected by a Co-IP assay. (B) HEK293T cells were cotransfected with Flag-SARS-CoV-2 S1 and Myc-Claudin-1 for 24 h. The cells were incubated with the mouse anti-Flag mAb and rabbit anti-Myc mAb. Fluorescence images were acquired with confocal microscopy. Bars, 5 μm.


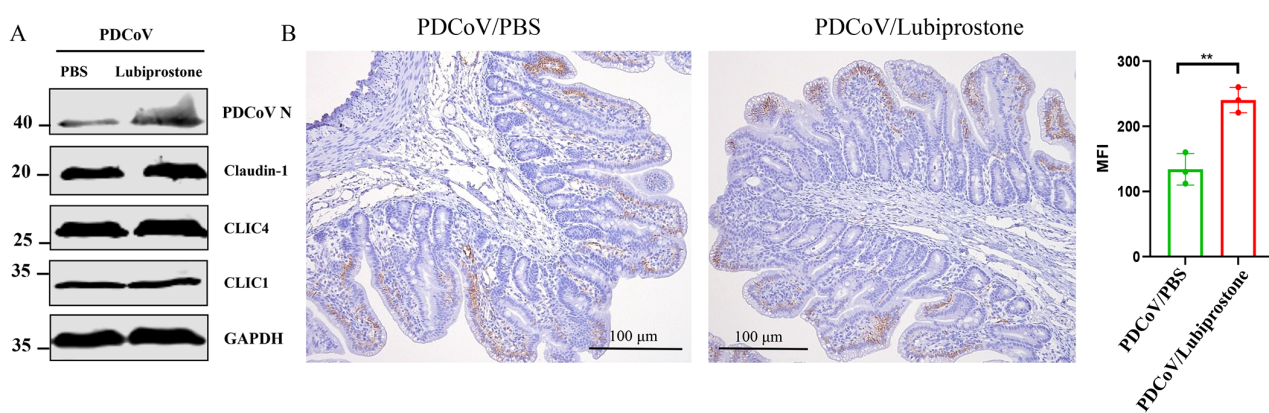


**Fig. S6. Lubiprostone treatment increased claudin-1 expression but does not affect the expression of CLIC1 and CLIC4.** (A) The expression of claudin-1, CLIC1, CLIC4 and PDCoV-N were assayed by Western blotting. (B) Immunohistochemical analysis was conducted to detect the expression of claudin-1 in jejunum tissues of PDCoV/PBS and PDCoV/Lubiprostone groups (Bars, 100 μm).
